# Recovery From Acute Kidney Injury With Diabetic Ketoacidosis Following SARS-CoV-2 Infection: A Case Report and Literature Review

**DOI:** 10.7759/cureus.11702

**Published:** 2020-11-25

**Authors:** Chang Xu, Umer Zia

**Affiliations:** 1 Medicine/Nephrology, James J. Peters VA Medical Center, Bronx, USA; 2 Medicine, Vassar Brothers Medical Center, Poughkeepsie, USA

**Keywords:** continuous renal replacement therapy (crrt), renal replacement therapy, diabetic ketoacidosis (dka), covid-19, kidney function recovery, acute kidney injurym

## Abstract

Diabetic ketoacidosis (DKA) is a life-threatening complication of type 1 diabetes (DM) and sometimes type 2 diabetes. DKA in COVID-19 patients predicts a poor prognosis. There are few published reports describing DKA and AKI in COVID-19 patients. There is even less information on renal recovery or follow up of these patients. We report a case of a 55-year-old man with type 2 diabetes who presented with cough and shortness of breath. He was found to have DKA, AKI, and COVID-19. He was admitted to the ICU and subsequently intubated for airway protection and started on renal replacement therapy for AKI. The patient’s renal function eventually recovered after 48 days of hospitalization, and he was discharged from the hospital. We report this case and performed a literature review to highlight that COVID-19 can lead to DKA and severe AKI in patients with DM. More importantly, we described the clinical and laboratory data that are associated with the recovery of COVID-19 AKI that are rarely reported.

## Introduction

In December 2019, coronavirus disease 2019 (COVID-19) was first reported in Wuhan, China, and rapidly became a global pandemic by March 2020. A recent study showed acute kidney injury (AKI) developed in 36.6% of patients hospitalized with COVID-19 in metropolitan New York. Of these, 14.3% required renal replacement therapy (RRT) [1]. Diabetic ketoacidosis (DKA) is a life-threatening complication of type 1 diabetes (DM) and sometimes type 2 diabetes. DKA in COVID-19 patients predicts a poor prognosis with a mortality rate of about 50% [2]. In the COVID-19 pandemic, it has been established that AKI is a frequent complication and is associated with increased mortality [3]. A recent cohort of hospitalized patients with COVID-19 showed that AKI occurs frequently among patients with COVID-19 disease especially in patients who require ventilation. The development of AKI in patients hospitalized for COVID-19 portends a poor prognosis. Diabetes mellitus is one of the risk factors for AKI in patients with diabetes compared to a nondiabetic population [1]. Here, we describe one patient with a history of type 2 diabetes who presented to our hospital with DKA, AKI, and severe acute respiratory distress syndrome caused by severe acute respiratory syndrome coronavirus 2 (SARS-CoV-2). We also reviewed almost all published cases of COVID-19-related DKA and AKI.

## Case presentation

A 55-year-old man presented to our hospital’s emergency room on April 12, 2020, with complaints of anorexia, cough, and shortness of breath. The patient had a seven-day history of cough, fever, myalgia, and malaise before presentation. His medical history included diabetes, diverticulitis, and hyperlipidemia. Home medications included atorvastatin, clomiphene, insulin Humalog 16 units subcutaneous before meal and bedtime, canagliflozin, Levemir, 54 units subcutaneous daily, lisinopril, and metformin. Baseline serum creatinine was 1.24 mg/dl on routine laboratory testing prior to admission. He had no family history of kidney disease. He did not use tobacco or illicit drugs.

In the emergency department (ED), the patient was afebrile, oral temperature 97.6, blood pressure 105 mmHg systolic and 58 mmHg diastolic, tachycardic heart rate (HR) 128, tachypneic respiratory rate (RR) 28, blood glucose level 559 mg/dL, arterial blood gas (ABG) revealed pH arterial 7.11, partial pressure of carbon dioxide (PCO_2_) arterial 16, PCO_2_ total arterial 6, HCO_3_ arterial 5.1, PCO_2_ arterial 66. Arterial blood gas findings in the setting of hyperglycemia were consistent with DKA. Findings were further confirmed by positive ketonuria on urinalysis (UA). He was also found to have lactic acid level of 4.2 mmol/L in the setting of regular metformin use at home. 

Initial blood and urinary examination are shown in Table 1. Renal function was abnormal (blood creatinine 2.96 mg/dL) and hematuria and proteinuria were detected. Complete metabolic panel also revealed serum sodium 124 mmol/L, potassium 7 mmol/L, bicarbonate 8 mmol/L, glucose 525 mg/dL, elevated anion gap of 31. Inflammatory markers were elevated (lactate dehydrogenase level 501 IU/L, C reactive protein 32.7 mg/L, ferritin level 2951 ng/mL, and procalcitonin level 4.31 mg/ml). The chest X-ray showed mild patchy bilateral pulmonary opacities. Patient was tested for COVID-19 and he tested positive.

**Table 1:** Laboratory Data CRRT: Continuous renal replacement therapy, HD: hemodialysis

Variable	Reference range, Adults	Prior to admission	On admission	Hospital Day 2	Hospital Day 3	Hospital Day 19	Hospital Day 20	Hospital Day 45	Hospital Day 48
Events				CRRT		Off CRRT	HD	Last HD	
Blood									
Hematocrit (%)	36-46	50.5	45.1	38.3	37.5	21.2	23.3	29.3	28.6
Hemoglobin (g/dl)	12-16	14.1	16	14.5	13.1	6.9	7.4	9.9	9.9
White cells (per ml)	4500-11000	5	9.7	6.8	5.9	4.4	5.9	10.7	10.0
Differential Count (per ml)									
Neutrophils Auto%	50-80	47	88.6	85.2	85.9	70.7	74.6	62.5	64.1
Lymphocytes Auto%	14-44	39.3	3.1	8.7	8.1	14.6	9.9	16.5	18.7
Monocytes Auto%	0-12	7.9	8.9	5.6	4.8	10.6	8.9	8.8	7.6
Eosinophils Auto%	0-7	4.6	0	0.2	0.1	6.1	10.1	10.9	8.6
Platelets	150,000-400,000	184	255	192	128	149	168	287	282
Prothrombin time (sec)	11.5-14.5		11.3	12.6	12.4	13.3	13.1	33.1	15.5
Prothrombin-time international normalized ratio	0.9-1.1		1.0	1.1	1.1	1.1	1.1	2.8	1.3
Activated partial-thromboplastin time ( sec)	22-35		21.5	23.1	61.8	57.1			
Sodium (mmol/liter)	135-145	137	124	138	138	133	132	133	133
Potassium (mmol/liter)	3.4-4.8	4.9	7	5.4	4.6	3.7	4.6	3.8	3.8
Chloride (mmol/liter)	100-108	99	85	103	102	100	100	94	94
Carbon dioxide (mmol/liter)	23.0-31.9	30	8	27	26	21	19	29	29
Urea nitrogen (mg/dl)	8-25	27	53.2	43.9	31.2	51.3	80.2	34.8	39.7
Creatinine (mg/dl)	0.6-1.5	1.24	2.96	2.62	1.83	3.44	5.23	1.89	1.38
Glucose (mg/dl)	70-110	161	525	191	279	108	210	224	216
D-dimer(ng/ml)	<500		4906	48619	33918	11015	12033	2064	
Ferritin (mg/liter)	20-300		2951	3026				1982	
Lactate dehydrogenase (U/liter)	110-210		501						
C-reactive protein (mg/liter)	<8		32.7	268.7				2.7	1.7
Lactic acid			4.2			1.3			
Procalcitonin	0-0.5 ng /ml		4.31					0.53	
Urine									
Bilirubin	Negative		Negative						Negative
Blood	Negative		Small (+1)						Negative
Clarity	Clear		Clear						Cloudy
Glucose	Negative		Positive (+3)						Positive (+2)
Ketones	Negative		Positive (+2)						negative
pH	5-9		5.0						5.5
Protein	Negative		Trace						Positive (+1)
Specific gravity	1.001-1.035		1.02						1.017
Red cells (per HPF)	0-2		10-20						0-2
White cell (per HPF)	<10		0-5						0-5

Clinical course

In the ED, the patient was hypoxic O_2_ saturation 85% on room air. The patient was started on nasal cannula oxygen with minimal improvement in saturation after which he was put on 15 L of high flow nasal cannula oxygen without any improvement in O_2_ saturation. Then the patient was given etomidate 20 mg IV, succinylcholine 150 mg IV and was intubated and put on ventilator support. The patient was then admitted to intensive care unit. The patient was found hypotensive 90/52 mmHg after intubation and was started on norepinephrine for hemodynamic support. The patient was given 10 units of insulin as bolus with maintenance insulin drip. He was also administered azithromycin 500 mg IV and ceftriaxone 1 g IV and was continued on broad spectrum antibiotics. 

Nephrology was consulted due to worsening kidney function, hyperkalemia, and decreased urine output. The patient was started on continuous renal replacement (CRRT) on hospital day two, about 27 hrs after admission. CRRT was provided as continuous venovenous hemodiafiltration using a Prismaflex CRRT machine (Baxter Inc, Deerfield, IL). The prescribed dose is 25 ml/kg/h. We used titratable anticoagulation with unfractionated heparin after CRRT was started. After a week on CRRT the patient continues to have hypoxia requiring prone positioning and mechanical ventilation. The patient was given convalescent plasma on hospital day six as an experimental treatment due to worsening respiratory status and multiorgan failure. On day nine, the patient continues to have minimal urine output, he was transfused convalescent plasma again, no remdesivir or tocilizumab was given during the hospitalization. The patient received dexamethasone since hospital day six for total eight days. The patient also had a thromboembolic event, he was found to have acute thrombosis of left common femoral vein, femoral and popliteal vein on hospital day 33, he remained on IV heparin which was later transitioned to coumadin. 

On hospital day 19, the patient remained intubated, off pressor, CRRT was discontinued, serum creatinine was 3.44 mg/dL on CRRT that increased to 5.23 mg/dL the next day. On hospital day 20, he was started on hemodialysis. He was continued on hemodialysis on Monday, Wednesday, and Friday schedule. Patient underwent tracheostomy on day 24. The patient was weaned off ventilator on day 30 and placed on trach collar. Prior to day 42 the patient remained oliguric. The patient since then started to make good urine output gradually >30 cc/hr and received last hemodialysis on day 45. On day 48, serum creatinine level improved to 1.30 mg/dL after he stopped dialysis since hospital day 45 (Table 1). He was tested for COVID 19 again on day 32 and test was repeated for next two days, all tests came back negative. The patient was eventually discharged to sub-acute rehabilitation after 48 days in hospital.

## Discussion

Here we present a 55-year-old man with severe acute kidney injury and DKA requiring renal replacement therapy in the context of COVID-19 illness.

How COVID-19 impacts people with diabetes is unclear, it has been shown that SARS-CoV-1 binds to the angiotensin-converting enzyme 2 (ACE2) receptor in the pancreatic islets, and it is postulated that this could cause pancreatic islets damage and lead to insulinopenia and increased risk of DKA, especially for those with established Type 2 DM [4]. Whether the new SARS-CoV-2 virus could cause similar pancreatic islets damage and increased the risk of DKA is not known. To date, only one study has described the prevalence of DKA in 658 hospitalized patients with confirmed COVID-19 [5]. Of the cohort, 42 (6.4%) out of 658 patients presented with ketosis on admission, three (7%) of those 42 patients met the American Diabetes Association criteria for DKA, the three individuals who developed DKA had underlying diabetes (one with Type 1 DM, two with Type 2 DM). Whether the three patients developed AKI and how they are managed was not described in this study. DKA has been reported in COVID-19 with no concurrent AKI [6]. Few publications described management of DKA and AKI in COVID-19 patients. One report presented the case of a COVID-19 patient who developed AKI and DKA. The patient was started on continuous renal replacement therapy the sixth day after admission and eventually died after the 16th day of hospitalization [7].

The etiology of acute kidney injury occurring in COVID-19 patient is not clear. Many studies reported proteinuria and microscopic hematuria in such patients. Our patient also has hematuria and low-grade proteinuria. Proposed mechanisms include direct viral-induced tubular or glomerular injury, altered renin-angiotensin-aldosterone (RAAS) regulation, rhabdomyolysis, and thrombotic disease [8,9]. Kidney biopsy findings include acute tubular necrosis [10], glomerular fibrin thrombi, pigmented tubular casts, and collapsing focal segmental glomerulosclerosis [9].

Proinflammatory cytokines (tumor necrosis factor α (TNFα), Interleukin 6 (IL-6)) and C-reactive protein (CRP) are also elevated in the setting of DKA independent of accompanying illness. Although, in DKA patients all these values reached near normal levels with insulin therapy and resolution of glycemic crises within 24 h, likely due to an anti-inflammatory effect of insulin [11].

Whether DKA and severe COVID-19 are synergistic in leading to severe AKI remains to be seen. Studies showed that increased inflammatory markers in serum of COVID-19 patients, especially CRP, procalcitonin, IL-6, and erythrocyte sedimentation rate (ESR), were positively correlated with the severity of COVID-19. In our patient, serum CRP, procalcitonin, and ferritin were all significantly elevated on admission (Table 1). Anti-inflammatory treatments include steroids, intravenous immunoglobulin, selective cytokine blockade inflammatory agents are currently being investigated to treat severe COVID-19 [12,13]. Steroid was used in our patient as described in clinical course. In this patient, we indeed found that the initially elevated serum proinflammatory marker level and D-Dimer levels on admission all decreased with treatment, by the time patient’s renal function improved on day 45, D-Dimer, CRP, ferritin, and procalcitonin level all decreased (Table 1). AKI and hematuria seen on admission also resolved on hospital day 48 with treatment (Table 1).

There have been significant developments in the management of COVID-19-associated acute kidney [14] since we treated this patient in April 2020. This patient was given dexamethasone 4 mg every six hrs (intravenous) since hospital day six for four days then 4 mg every 12 hrs for another four days instead of 6 mg once daily for 10 days as recommended by the University of Oxford’s RECOVERY trial, which published preprint paper on the effect of dexamethasone on COVID-19 in June 2020. Recovery trial showed that dexamethasone reduced 28-day mortality among hospitalized patients with COVID-19, especially among patients receiving either mechanical ventilation or oxygen therapy alone [15]. Extracorporeal blood purification treatment including plasmapheresis has been used to remove inflammatory cytokines that potentially cause hyperinflammation and hypercoagulability in critically ill patients with COVID-19. No sufficient research data and consensus exists on the use of extracorporeal blood purification treatment in critically ill patients with COVID-19 [14].

## Conclusions

In summary, in this article we described a patient with severe AKI in the setting of COVID-19 pneumonia and DKA who was treated with CRRT initially. The patient's renal function eventually returned to normal after 48 days. To the best of our knowledge, this is one of the few reports describing the clinical and laboratory data leading to the recovery of severe COVID-19 AKI associated with DKA.
